# A Quad-Band and Polarization-Insensitive Metamaterial Absorber with a Low Profile Based on Graphene-Assembled Film

**DOI:** 10.3390/ma16114178

**Published:** 2023-06-04

**Authors:** Shiyi Jin, Haoran Zu, Wei Qian, Kaolin Luo, Yang Xiao, Rongguo Song, Bo Xiong

**Affiliations:** 1 School of Science, Wuhan University of Technology, Wuhan 430070, China; 305863@whut.edu.cn (S.J.); qianwei@whut.edu.cn (W.Q.); 261162@whut.edu.cn (K.L.); 261206@whut.edu.cn (Y.X.); 2 Hubei Engineering Research Center of RF-Microwave Technology and Application, Wuhan University of Technology, Wuhan 430070, China; zuhr@foxmail.com

**Keywords:** metamaterial absorber, quad-band, graphene-assembled film, low profile, polarization-insensitivity

## Abstract

A quad-band metamaterial absorber using a periodically arranged surface structure placed on an ultra-thin substrate is demonstrated in this paper. Its surface structure consists of a rectangular patch and four L-shaped structures distributed symmetrically. The surface structure is able to have strong electromagnetic interactions with incident microwaves, thereby generating four absorption peaks at different frequencies. With the aid of the near-field distributions and impedance matching analysis of the four absorption peaks, the physical mechanism of the quad-band absorption is revealed. The usage of graphene-assembled film (GAF) provides further optimization to increase the four absorption peaks and promotes the low-profile characteristic. In addition, the proposed design has good tolerance to the incident angle in vertical polarization. The proposed absorber in this paper has the potential for filtering, detection, imaging, and other communication applications.

## 1. Introduction

Metamaterials are artificial subwavelength composite structures with unusual material properties. They play an important role at the forefront of research in electromagnetism, optics, engineering, and materials science today [1]. Due to their superior ability to control electromagnetic waves [2,3,4], metamaterials have promising applications in sensors [5,6], antennas [7,8], super lenses [9,10], holographic surfaces [11,12], radar scattering cross-section reduction [13,14], etc. Metamaterial absorbers, as an important branch of the metamaterial device field, have attracted extensive research interest in civil and military fields, as they have many advantages such as near-perfect absorption, a low profile, and flexible options for absorption frequencies. The thickness of metamaterial absorbers is only a few tenths or even one-hundredth of the resonance wavelength, which makes them have the advantages of being lightweight, compact, and easy to construct. They have been demonstrated to be promising for a wide range of applications in terms of thermal imaging, bolometers, optical switches, refractive index sensors, solar cells, stealth technology, and so on [15,16]. However, metamaterial absorbers have usually exhibited single-band absorption ever since the first metamaterial absorber operating in the microwave region was reported [17], which greatly limits their application prospects.

It would broaden the application potential of metamaterial absorbers to achieve near-perfect absorption in multiple frequency bands in a selected spectral range; therefore, multi-band absorption is necessary and worth studying. Usually, the stacking method [18,19] and co-planar design strategy [20,21,22] are commonly used in the designing of multi-band absorbers; they can both increase the number of absorption peaks by arranging multiple resonators with different dimensions or shapes. However, these methods have many disadvantages, such as their complex structure design and high profile. More importantly, every layer needs to be accurately aligned— otherwise, the designed resonant structures cannot support multi-band absorption. Placing too many resonator structures within the same layer usually results in large unit dimensions, which cannot fit current design trends towards miniaturization. Instead of these two current methods, using novel conductive materials such as two-dimensional graphene instead of metals with a fixed, ultra-high conductivity could be a new way to achieve multi-band absorption [23]. Such methods cannot only partly solve the problems mentioned before, but could also improve the corrosion resistance and environmental protection of electronic devices. However, the production technology for 2D graphene is limited—thus, it cannot be prepared in large quantities, and so most research results are still at the simulation design stage. Furthermore, its conductivity is too low within the microwave band, and so cannot produce resonation.

Recently, a graphene-assembled film (GAF) with customizable electrical conductivity has been widely used instead of metals in the designing of microwave devices. Graphene is arranged in a honeycomb pattern by single-layer carbon atoms, with novel properties such as high electron mobility, mechanical flexibility, and optional conductivity. The conductivity of GAF can be as high as 1.1 × 10^5^ S/m and above, which is sufficient to meet the requirements of RF electronic devices [24,25]. What is more, its optional conductivity can broaden the range of design ideas for multi-band metamaterial absorbers, which could efficiently solve the problems mentioned above. Furthermore, GAF’s ultra-thin and flexible characteristics can also greatly reduce the difficulty of device preparation [26,27]. GAF has excellent high conductivity, flexibility, corrosion resistance, and high-temperature resistance [28,29,30], which make it an excellent material for making metamaterial absorbers.

In this work, we investigate and propose a method to increase the number of absorption peaks of a metamaterial absorber. First, this work proposes a quad-band metamaterial absorber—the top structure of which consists of a rectangular patch and four L-shaped structures arranged in a regular pattern. The structure can interact with the incident wave and excite four discrete resonant peaks in the microwave band, but the absorption efficiency of all four resonant peaks cannot reach 90%. Then, GAF with a conductivity of 1.1 × 10^5^ S/m has been used instead of copper to fabricate the top structure. The results show that the use of GAF can effectively increase the absorption efficiency—thus increasing the number of absorption peaks. The proposed metamaterial absorber can provide four absorption peaks at 4.70, 10.18, 11.96, and 14.15 GHz, covering the C, X and Ku-bands. As such, it can be applied for short-range microwave sensing and satellite and radar communications. Subsequently, this paper discusses the effects of variations in top-structure parameters on the performance of the metamaterial absorber; this could help in the optimization of these parameters to obtain an optimal structure. In addition, this work analyses the physical mechanisms of quad-band absorption with the help of the impedance matching theory and the surface current distribution at the four absorption peaks. Our research aims to achieve improvements in the absorption efficiency of metamaterial absorbers by the simple method of using GAF, which can not only exhibit better properties such as enhanced absorption amplitude and reduced weight, but also reduce costs. In addition, this work verifies that GAF can be applied to metamaterial absorbers and even other metamaterial research areas.

## 2. Characterization of Graphene-Assembled Film

**Figure 1:**
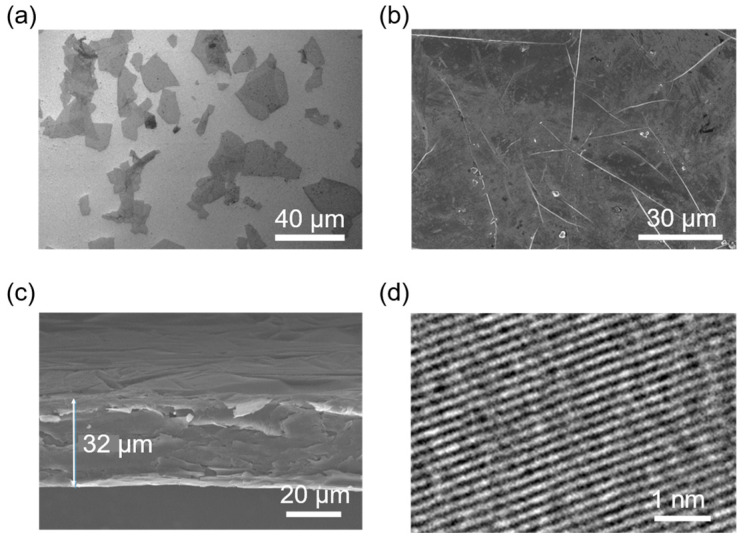
(**a**) SEM image of the GAF nanosheet. (**b**) Surface SEM image of the GAF. (**c**) Cross-sectional SEM image of the GAF. (**d**) HRTEM image of the GAF.

The conductivity of the GAF was 1.1 × 10^5^ S/m, as measured by the Four-Probe method. Figure 1a,b shows the scanning electron microscopy (SEM; JEOL JEM6700, Tokyo, Japan) images of the GAF nanosheet and the surface of the GAF, from which it can be seen that there are a large number of micro-folds on the surface of the GAF. These micro-folds are attributed to the synergistic effect of graphene nanosheet stacking. Due to the existence of these folds, GAF has excellent flexibility, as it has great buffering and unloading force when subjected to external forces. Figure 1c is the cross-sectional SEM image of the GAF, which shows the GAF with a thickness of 32 μm has a regular stacking structure. The high-resolution transmission electron microscope (HRTEM, JEOL JEM-2100F, Tokyo, Japan) image of the GAF in Figure 1d shows the regular lattice fringe of the GAF nanosheets and shows that it was highly graphitized.

**Figure 2:**
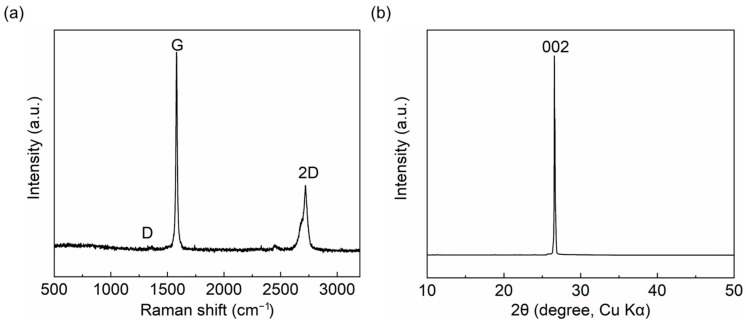
(**a**) The Raman spectrum of the GAF. (**b**) The XRD pattern of the GAF.

In Figure 2a, the Raman spectrum of the GAF shows a small D (1344 cm^−1^) peak and a sharp G (1582 cm^−1^) peak, which proves hat GAF has fewer lattice defects and high conductivity. As shown in Figure 2b, the X-ray diffraction (XRD) pattern shows two characteristic graphitic peaks locate at 2θ = 26.5° (002) and 2θ = 54.7° (004), respectively. The two characteristic graphitic peaks indicate that the interlayer spacing of the graphene layer is 0.34 nm, as well as the high graphitization structure of the GAF.

## 3. Methods

**Figure 3:**
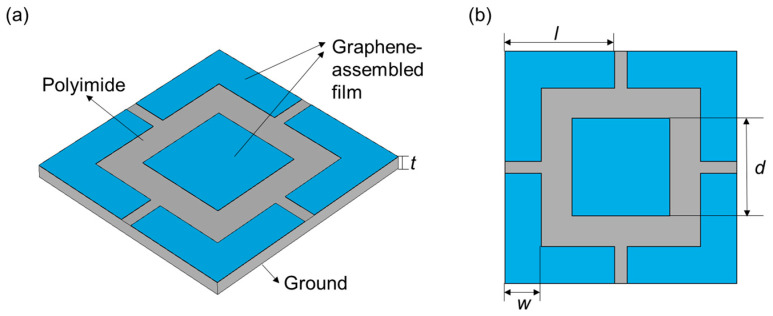
Side view (**a**) and vertical view (**b**) of the quad-band metamaterial absorber.

The basic unit cell of the proposed quad-band metamaterial absorber consisted of a top surface structure, a dielectric spacer layer, and a ground plane—as shown in Figure 3a. The surface structure of the quad-band metamaterial absorber consisted of a rectangular patch and four symmetrically distributed L-shaped structures with equal arm lengths—as shown in Figure 3b—where the periodicity was *p* = 19 mm, the length of the side of the rectangular patch was *d* = 7 mm, the L-shaped structure arm length was *l* = 9 mm, and the width of L-shaped structure was *w* = 3 mm. The thickness of the dielectric layer *t* = 0.8 mm was about 1/77 of the resonant wavelength of the first absorption peak (4.7 GHz), which meets the values for the low-profile characteristic. A lossy material polyimide (PI) with a dielectric constant set to 3.5 was used as the dielectric layer.

By adjusting the surface impedance of the designed metamaterial structure, the surface impedance (Z_r_) can be equivalent to the wave impedance in the air (Z_0_ = 377 Ω). Thus, the reflectance of the absorber structure can approach approximately zero, where Z_r_ = (*μ*_0_ *μ*_r_/*ε*_0_ *ε*_r_)^1/2^, Z_0_ = (*μ*_0_/*ε*_0_)^1/2^. The simultaneous suppression of transmission and reflection provides the possibility of successfully achieving near-perfect absorption, which can be mathematically expressed as:

A(ω) = 1 − R(ω) − T(ω) = 1 − R(ω)(1)

where A(ω) is the absorptivity, R(ω) is the reflectance, and T(ω) is the transmittance.

According to the equation of impedance Zr = r + jX = *R* + jω*L* − j/(ω*C*), the resistance can also change the impedance of the metamaterial absorber to match or mismatch with the free space impedance. This fully illustrates that the absorption efficiency at each operating frequency can be improved by changing the square resistance of the surface of the metamaterial absorber. Based on the relationship between the square resistance and conductivity—*R*_s_ = 1/*d*σ—it can be seen that the GAF, which possesses a conductivity different from metals, is an excellent material to achieve this function.

The proposed structure was simulated by CST Microwave Studio software based on a frequency domain solver. The frequency range of the frequency domain solver was from 4.0 to 16.0 GHz. S_11_(ω) and S_21_(ω) in the simulation results were the reflection and transmission coefficient, respectively, so that the absorption rate of the metamaterial absorber can be expressed as:

A(ω) = 1 − |S_11_(ω)|^2^ − |S_21_(ω)|^2^ = 1 − |S_11_(ω)|^2^ (2)

## 4. Results and Discussion

**Figure 4:**
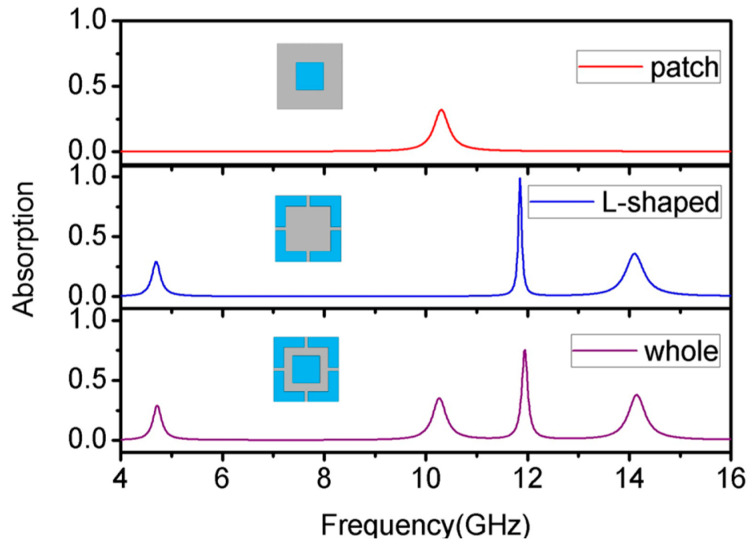
The simulated absorption spectra of each structure.

This part uses copper as the conductive material of the top structure for the simulation to introduce the design process and the cause of the excitation of each absorption peak. The simulated absorption spectra of the patch structure, the L-shaped structure, and the overall structure—with the combination of both—are given in Figure 4. The results show that the patch structure can produce an absorption peak with an absorptivity of 52.25% at 10.28 GHz, and the L-shaped structure can produce three absorption peaks with an absorptivity of 53.06%, 94.80%, and 54.45% at the three central frequency points of 4.70 GHz, 11.92 GHz, and 14.28 GHz, respectively. Integrating these two structures onto the same surface, the proposed metamaterial absorber can yield 39.87%, 45.21%, 85.39%, and 48.34% absorptivity at 4.70 GHz, 10.19 GHz, 11.96 GHz, and 14.15 GHz, respectively.

**Figure 5:**
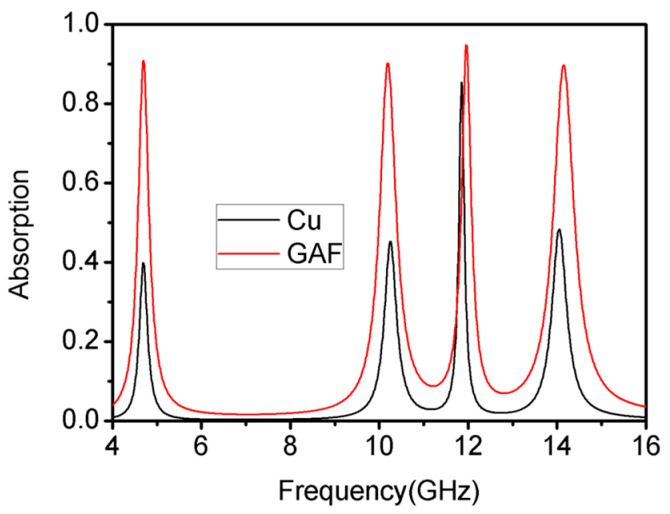
Comparison of absorption characteristics of metamaterial absorbers with different materials (graphene and copper).

It is obvious that although the copper metamaterial absorber can form multiple resonance peaks, the absorption efficiency of three resonance peaks cannot reach 90%. The GAF used in this paper had a conductivity of 1.1 × 10^5^ S/m, which can be characterized by setting a material of lossy-type metal with a conductivity of 1.1 × 10^5^ S/m and a thickness of 0.032 mm in CST. The comparison is shown in Figure 5. The result shows that the absorption efficiency of all four absorption peaks was improved significantly, while the change in absorption frequency was minimal. The absorption efficiency of the four absorption peaks increased from 39.87%, 45.21%, 85.39%, and 48.34% to 90.89%, 90.18%, 94.80%, and 90.02%, respectively. It can be seen that the metamaterial absorber based on the GAF was easier to achieve higher absorption with than the conventional metallic metamaterial absorber.

**Figure 6:**
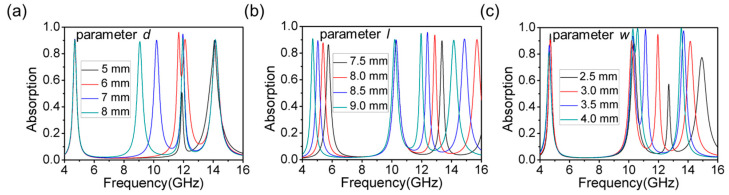
Effect of changing (**a**) parameter *d* (**b**) parameter *l* or (**c**) parameter *w* on the absorption characteristics of metamaterial absorbers.

Subsequently, Figure 6 shows the effect of each parameter of the structure on the absorption spectrum of the absorber. The simulation results showed that changes in the structure parameters can lead to variations in both the frequency and amplitude of the absorption peaks. Figure 6a presents the effect of the patch edge length *d*. When only parameter *d* is changed, the absorption efficiency and absorption frequency of the first and fourth absorption peaks hardly changed, whereas the changes in the second and third absorption peaks were obvious. When *d* decreased, the frequency of the second absorption peak moved from high to low frequencies. When *d* = 6 mm, the second absorption peak moved to 11.68 GHz with a partial overlap with the third absorption peak. When *d* = 5 mm, the second absorption peak disappeared and the absorption efficiency of the third absorption peak was visibly reduced, so that the absorber had only two effective absorption peaks. To ensure the absorption efficiency of the four absorption peaks, we determined *d* = 7 mm to be the final optimization result.

Next, Figure 6b shows how parameter *l* affected the absorption characteristics of this absorber. Changes in parameter *l* had little effect on the second absorption peak. For the other three absorption peaks, the decrease in *l* led to a shift to a higher frequency. In terms of the absorption efficiency, the first absorption peak gradually decreased from 90.07% to 86.36%, the third absorption peak first increased to 95.61% and then decreased to 88.43%, and the fourth absorption peak remained unchanged. As such, it is necessary to make the l value as large as possible. Combined with the actual processing of the minimum accuracy of 1 mm, there is no doubt that *l* = 9 mm was the best result.

Finally, Figure 6c shows the effect of the line width *w* of the L-shaped structure on the absorption performance of this absorber. For the first and the second absorption peaks, changes in *w* had almost no effect on the absorption frequency and only a small effect on the absorption efficiency. As the value of *w* increased, the absorption efficiency of the first and second absorption peaks gradually decreased and increased, respectively. The frequency and efficiency of both the third and fourth absorption peaks changed more significantly. The efficiency of the third absorption peak increased from 57.28% to 99.51%, and the frequency shifted from 12.7 GHz to 10.59 GHz. Meanwhile, the efficiency of the fourth absorption peak increased from 77.42% to 99.87%, and the frequency shifted from 14.93 GHz to 13.54 GHz. However, when *w* increased, the third absorption peak gradually approached the second absorption peak; in particular, when *w* = 4 mm, the two absorption peaks overlapped and were almost fused into one absorption peak. Therefore, to keep a certain distance between the second and third absorption peaks to avoid mutual interference, a *w* value of 3 mm is the best choice.

The central frequency of the first absorption peak was only sensitive to the *l* parameter. The central frequency decreased by about 0.8 GHz when *l* increased by 0.5 mm. The central frequency of the second absorption peak was only affected by parameter *d*. Thus, it could be adjusted by controlling parameter *d*. The third and fourth absorption peaks were both affected by parameter *l* and parameter *w*, and the influence trend was roughly the same. Parameter *l* only changed the absorption frequency of the two absorption peaks, while parameter *w* changed both the central frequency and the absorption efficiency of the two absorption peaks.

**Figure 7:**
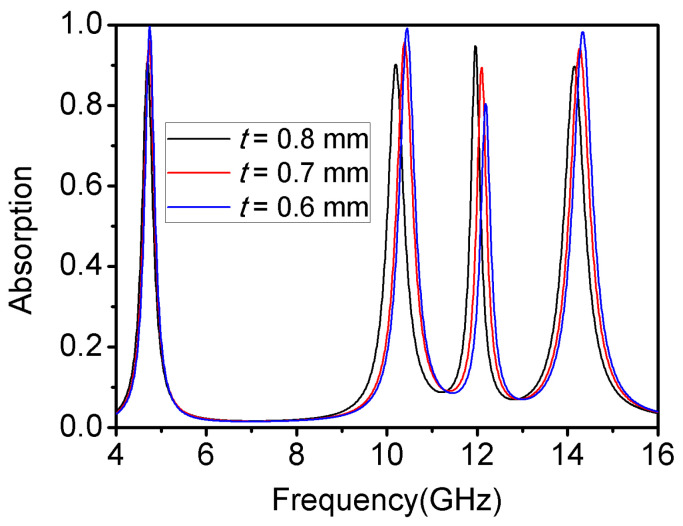
Influence of thickness *t* on the absorption characteristic.

In order to make the profile of the absorber as low as possible, it was necessary to explore how the thickness of the dielectric layer influenced the absorption characteristics of the proposed absorber. As shown in Figure 7, decreasing the thickness *t* can increase the absorption efficiency and bandwidths of the first, second, and fourth absorption peaks. However, the absorption efficiency of the third peak decreased visibly to less than 90%. In this case, the absorber could not meet the requirements of the quad-band metamaterial absorber. As such, a thickness of 0.8 mm is the thinnest possible on the premise of meeting the requirements of the quad-band characteristic.

**Figure 8:**
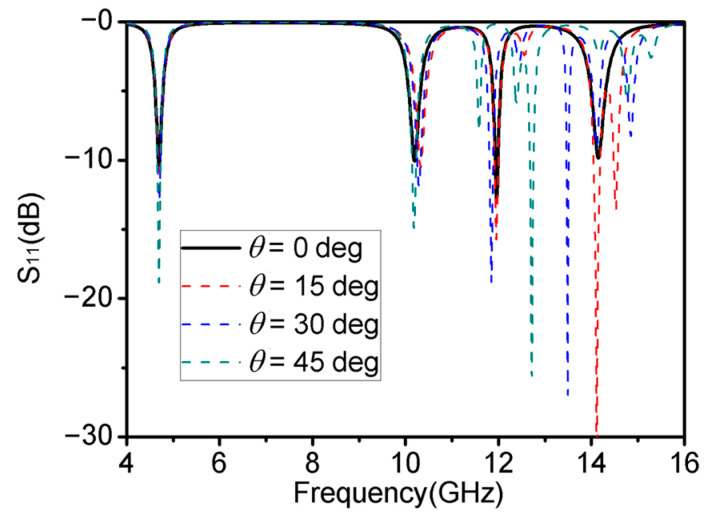
Absorption characteristics of the TE waves created by the multi-band metamaterial absorbers at different incidence angles *θ*.

Given that most of the antenna polarization methods use vertical polarization (TE mode) nowadays, Figure 8 shows the absorption characteristics of this multiband metamaterial absorber for TE waves at different incidence angles *θ*. Using the reflection coefficient S_11_ can show changes in absorption characteristics better. The results show that during the growth of *θ* from 0° to 30°, the reflection coefficients of the quad-band metamaterial absorber at the four resonant frequencies were all below −10 dB. When *θ* = 15°, the absorber added an absorption peak at 14.53 GHz with a reflection coefficient of −13.26 dB, corresponding to 95.28% absorption efficiency. When *θ* = 30°, the absorber added two absorption peaks at 13.49 GHz and 14.83 GHz with reflection coefficients of −26.96 dB and −8.02 dB, corresponding to 99.80% and 84.22% of the absorption efficiency. When *θ* was rotated to 45°, the absorber maintained its high absorption performance at the first and second peaks. However, the absorption peaks at the third and fourth frequency bands shifted to 12.72 GHz with a reflection coefficient of −25.60 dB, and some high harmonics were generated at high frequencies.

This quad-band metamaterial absorber was able to maintain stable absorption for electromagnetic waves in TE mode at four working frequencies when the incident angle was varied from 0° to 30°. It was also able to maintain stable absorption at the three frequency points of 4.70 GHz, 10.19 GHz, and 11.96 GHz when the incident angle was varied in the range of 0–45°. This illustrates that the proposed absorber showed good stability for oblique incidence.

**Figure 9:**
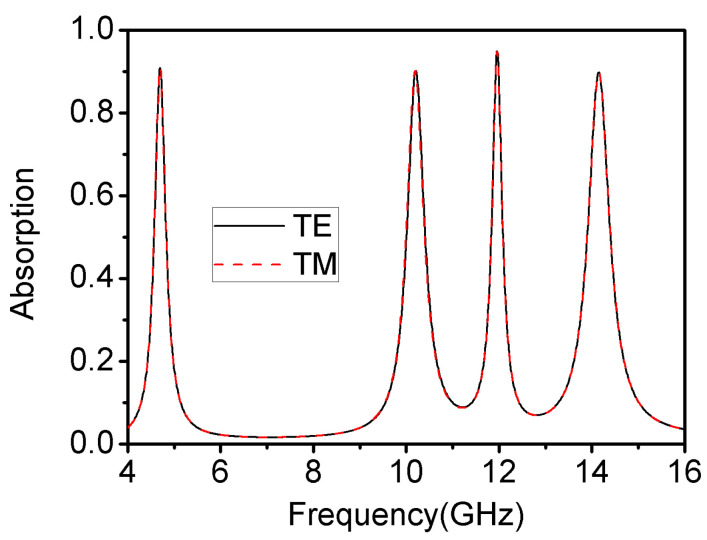
The absorption characteristics towards TE and TM polarized waves of the metamaterial absorber.

Figure 9 shows the absorption characteristics towards the TE and TM polarized waves of the proposed metamaterial absorber under vertical incidence. It can be seen that this absorber had the same response to both TE and TM waves under vertical incidence, illustrating the superior polarization insensitivity of this metamaterial absorber.

To explain why the proposed metamaterial absorber with GAF absorbed incident waves more efficiently than the conventional copper metamaterial absorber, this paper performed an impedance matching analysis of the two metamaterial absorbers. In order to achieve low reflection, it is necessary to make the input impedance Z_in_ equal to the impedance of free space Z_0_ as much as possible. Here, the complex impedance of the proposed metamaterial absorber was extracted by the inversion method [31]. The correspondence between the S-parameters and the complex impedance can be expressed by Equation (3):

(3) $$Z_{\mathrm{in}}=\pm \sqrt{\frac{{\left(1+S_{11}\right)}^{2}-{S_{12}}^{2}}{{\left(1-S_{11}\right)}^{2}-{S_{12}}^{2}}}$$

**Figure 10:**
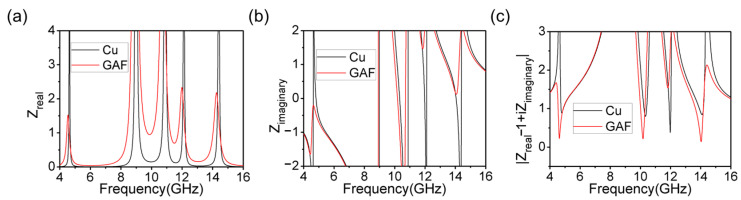
Impedance matching comparison between GAF and Cu. (**a**) Real part of the impedance. (**b**) Imaginary part of the impedance. (**c**) The formula describing the degree of impedance matching.

From Figure 10, the real and imaginary parts of the complex impedance fluctuated around 1 and 0 in the resonant frequency of the proposed metamaterial absorber; it was very close to the complex impedance in free space. An equation |(Z_real_ − 1) + iZ_imag_| can represent the absolute difference between the complex impedance of the proposed metamaterial absorber and free space. Obviously, the closer the value of |(Z_real_ − 1) + iZ_imag_| is to zero, the greater the complex impedance of the metamaterial absorber matches the free-space impedance—then, more electromagnetic waves can be transmitted to the dielectric layer, and the absorber can realize a higher absorption efficiency. It can be seen that the proposed GAF-based metamaterial absorber had a more perfect impedance-matching property than the copper metamaterial absorber; thus, it can be well explained why the metamaterial absorber based on GAF was able to achieve a higher absorption efficiency.

**Figure 11:**
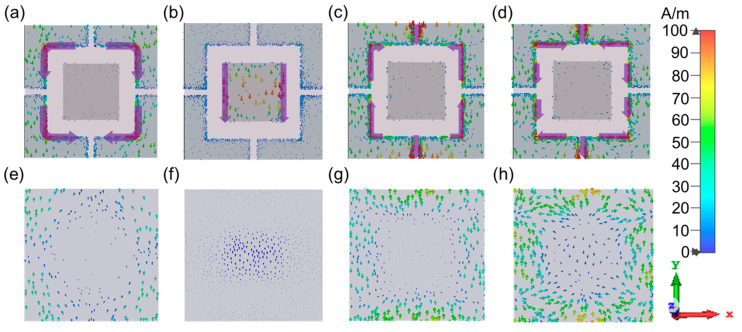
Surface current distribution. (**a**) Top layer structure 4.70 GHz. (**b**) Top layer structure 10.19 GHz. (**c**) Top layer structure 11.96 GHz. (**d**) Top layer structure 14.15 GHz. (**e**) Metal bonding layer 4.70 GHz. (**f**) Metal bonding layer 10.19 GHz. (**g**) Metal connection formation 11.96 GHz. (**h**) Metal connection formation 14.15 GHz.

In order to elucidate the mechanisms of the electromagnetic wave absorption of the metamaterial absorber more clearly, the surface current distribution on the top structure and the bottom grounding layer is given in Figure 11. Here, (a)–(d) are the current distributions of the top structure at four central frequencies of 4.70 GHz, 10.19 GHz, 11.96 GHz, and 14.15 GHz, and (e)–(h) are the current distributions of the bottom grounded metal layer at the same frequencies. It can be seen that at 10.19 GHz, the induced currents were mainly concentrated on the rectangular patch; this was a magnetic resonance caused by the reverse parallel induced surface currents on the top layer and the bottom layer. The surface current distribution also explains why the patch edge length *d* was the parameter that had the greatest effect on the second absorption frequency. Meanwhile, at 4.70 GHz, 11.96 GHz, and 14.15 GHz, the induced currents were mainly distributed on the L-shaped structure, indicating that the L-shaped structure played a major role at the first, third, and fourth absorption peaks. However, the current distribution of the L-shaped structure varied at different frequencies. At 4.70 GHz, the currents were mainly located on the two right-angled edges of the L-shaped structure with the same current direction. Meanwhile, the current direction on the top layer was opposite to that of the grounding layer; this indicates that the resonance at this frequency was provided by a combination of the two. At 11.96 GHz, the currents were strongly excited only on the right-angle and short sides of the L-shaped structure, with the same direction electric field, and the electric length was shorter compared to the first frequency. Furthermore, there was a current distribution in the opposite direction on the bottom grounding layer at the corresponding position. At 14.15 GHz, the currents were mainly distributed on the two right-angled edges and the short edges, with the same electric field direction. The currents on the two right-angled edges were in opposite directions.

The simulation results showed that changes in the L-shaped structure affected the positions of the first, third, and fourth operating frequencies in the absorption spectrum. The arm length *l* affected all of these three frequencies, while the line width *w* had a more significant effect on the third and fourth frequencies. This is in general agreement with the results discussed in the previous section for the absorption characteristic curves, which confirms the reliability of the experimental data.

**Figure 12:**
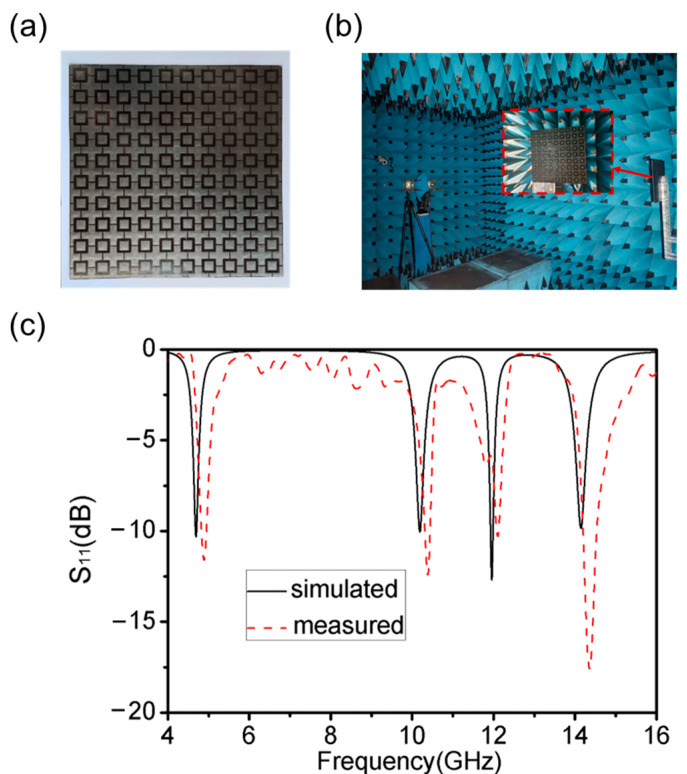
(**a**) Photograph of the metamaterial absorber sample. (**b**) Measurement darkroom schematic. (**c**) Quad-band metamaterial absorber reflection coefficient measured results.

The absorber sample used during the experiment is shown in Figure 12a. The top layer was prepared by etching the GAF attached to the polyethylene terephthalate (PET) film by a laser engraving machine and manually tearing off the excess [32], adhering it to the PI dielectric layer, and finally pasting another GAF onto the bottom layer to reduce the transmittance to nearly zero. Both GAF and PI have high thermal stability and corrosion resistance [26,27]. Thus, the absorber had superior environmental stability. The reflection parameters of the sample were measured using a vector network analyzer (VNA) ZNB20 connected to a pair of transmitter and receiver horn antennas—as shown in Figure 12b. The distance between the antennas and the sample was about 3 m.

The measured results showed a similar pattern to the simulated results. The GAF had a lower conductivity compared to metallic copper, and so it had a higher resistance value, which made it play the role of a resistive layer in the whole absorber. The resonator and the resistive layer were integrated into the same surface, which not only greatly reduced the processing difficulty and fabrication cost, but also effectively reduced the thickness of the absorber to achieve the low-profile characteristic. As can be seen from Figure 12c, using GAF with a conductivity of 1.1 × 10^5^ S/m to fabricate the top layer can help the reflection coefficients at all four central frequency points remain below −10 dB. The measured results show that this is a realistic and feasible way to improve the absorption efficiency of the absorber by changing the electrical conductivity of the top layer structure. This provides guidance for future work.

**Figure 13:**
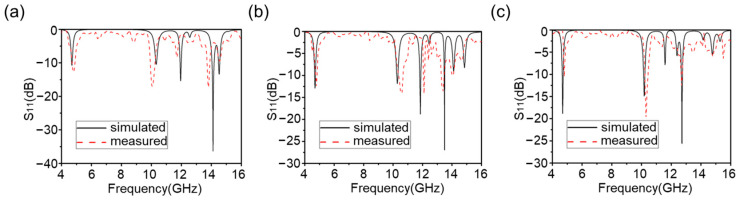
Reflection coefficients of multiband metamaterial absorbers at different incidence angles *θ* (**a**) *θ* = 15°, (**b**) *θ* = 30°, and (**c**) *θ* = 45°.

Next, the reflection coefficients of TE waves at different incidence angles *θ* were measured in this work—as shown in Figure 13. The measured results were almost consistent with the simulation results. During the growth of *θ* from 0° to 30°, the absorber was still able to achieve stable and effective absorption at the four central frequencies, despite the appearance of some higher harmonics in the high-frequency part. When *θ* grew to 45°, the absorption at the first and second frequency points could be maintained above 90%, and the absorption at the third and the absorption in the fourth band was not obvious. The measured results show that this quad-band metamaterial absorber exhibited oblique incidence stability within a certain angle, indicating that it has good environmental adaptability to realistic and variable application scenarios.

In this experiment, the size of the fabricated metamaterial absorber was 19 mm × 19 mm × 0.83 mm, which achieved good measurement results. The larger the size of the absorber, the better the absorption characteristic is, because it is closer to the infinite boundary conditions of the periodic structure. Therefore, the absorber can be integrated into satellites, radar communication, radomes, and other large equipment. In addition, graphene-assembled film (GAF) and PI film have the characteristic of flexibility, making it easy for them to conform with the equipment. Thus, the proposed metamaterial absorber in this paper has good scalability and integration potential.

A detailed comparative study was performed on the proposed metamaterial absorber vs. existing structures, as shown in Table 1. Different parameters were considered, such as thickness, absorption efficiency, and polarization insensitivity. As discussed in previous works, a metamaterial absorber that exhibits multiple absorption bands is preferable. The works in [33,34,35] have good performance in absorption efficiency, but they are not polarization-insensitive. The works in [36,37,38,39,40] have good polarization insensitivity; however, they perform less well in terms of having a low profile and their absorption efficiency. This article represents a low-profile, polarization-insensitive quad-band metamaterial absorber, which shows four absorption peaks in the C, X, and Ku-band frequencies. The proposed metamaterial absorber in this paper exhibits a good absorption performance for different values of permittivity.

## 5. Conclusions

A quad-band metamaterial absorber operating in the radar band is proposed and fabricated in this paper. The top pattern layer consisting of a rectangular patch and four L-shaped structures was prepared by replacing the metal with GAF with a conductivity of 1.1 × 10^5^ S/m. Compared with copper, this can help the absorber achieve more than 90% absorption at 4.70 GHz, 10.19 GHz, 11.96 GHz, and 14.15 GHz, respectively. Furthermore, the effective bandwidths were 30 MHz (4.68 to 4.71 GHz), 20 MHz (10.17 to 10.19 GHz), 60 MHz (11.93 to 11.99 GHz) and 12 MHz (14.14 to 14.152 GHz). The overall thickness of this metamaterial absorber was 0.83 mm, which shows a low-profile characteristic. Near-field distributions and impedance matching analysis of the four absorption peaks helped reveal the physical mechanisms of the quad-band absorption. The simulation and measurement results of the scattering parameter obtained from the TE mode matched well. Meanwhile, the metamaterial absorber had stable absorption at an incidence angle in the range of 0°–30° in vertical polarization. Our study provides a simple and low-cost method to increase the number of absorption peaks of multi-band metamaterial absorbers, which has potential for a variety of electromagnetic wave absorption devices such as filters, gas sensors/detectors, and energy, and also provides prospective validation for the study of conductivity-designable metamaterials.

## Figures and Tables

**Table 1:** Comparison table.

Ref.	Thickness (mm) (Corresponding to λ_0_)	Num. of Absorption Peaks	Frequency (GHz)	Absorption Efficiency	Polarization Insensitivity	Material
[33]	1.6 (0.036λ_0_)	4	6.8, 8.24, 11.23, 12.7	97%, 95%, 97%, 98%	No	Metal
[34]	1.5 (0.0112λ_0_)	4	2.248, 2.878, 4.3, 5.872	96%, 93%, 93%, 95%	No	Metal
[35]	1.57 (0.022λ_0_)	4	4.20, 10.14, 13.15, 17.1	97.9%, 99.5%, 99.1%, 99.95%	No	Metal
[36]	1.6 (0.066λ_0_)	4	12.4, 14.11, 17.56, 20.1	98.4%, 97.6%, 93%, 96.6	Yes	Metal
[37]	1.2 (0.0512λ_0_)	2	12.8, 15.5	90.5%, 90.3%	Yes	Metal
[38]	1.58 (0.059λ_0_)	4	11.23, 14.18, 17.37, 19.18	85.5%, 99.13%, 98.19%, 90.8%	Yes	Metal
[39]	1.58 (0.059λ_0_)	4	11.31, 14.11, 14.23, 17.79	94.63%, 95.58%, 97%, 75.58%	Yes	Metal
[40]	1.67 (0.0378λ_0_)	3	6.80, 8.36, 8.8	92.2%, 75%, 70%	Yes	Metal and Graphene ink
This work	0.83 (0.013λ_0_)	4	4.70, 10.19, 11.96, 14.15	90.89%, 90.18%, 94.80%, 90%	Yes	GAF

λ_0_ is the free-space wavelength with respect to the lowest resonance frequency (4.70 GHz).

## Data Availability

Not applicable.

## References

[1] Liu Y., Zhang X. (2011). Metamaterials: A New Frontier of Science and Technology. Chem. Soc. Rev..

[2] Veselago V.G. (1968). The Electrodynamics of Substances with Simultaneously Negative Values of ε and μ. Phys.-Uspekhi.

[3] Pendry J.B., Holden A.J., Stewart W.J., Youngs I. (1996). Extremely Low Frequency Plasmons in Metallic Mesostructures. Phys. Rev. Lett..

[4] Pendry J.B., Holden A.J., Robbins D.J., Stewart W.J. (1999). Magnetism from Conductors and Enhanced Nonlinear Phenomena. IEEE T. Microw. Theory.

[5] Bui T.S., Dao T.D., Dang L.H., Vu L.D., Ohi A., Nabatame T., Lee Y.P., Nagao T., Hoang C.V. (2016). Metamaterial-enhanced Vibrational Absorption Spectroscopy for the Detection of Protein Molecules. Sci. Rep..

[6] Cao Y., Ruan C., Chen K., Zhang X. (2022). Research on a High-Sensitivity Asymmetric Metamaterial Structure and its Application as Microwave Sensor. Sci. Rep..

[7] Porter R. (2020). Plate Arrays as a Perfectly-Transmitting Negative-Refraction Metamaterial. Wave Motion.

[8] Yang J., Chen S.T., Chen M., Ke J.C., Cui T.J. (2020). Folded Transmitarray Antenna with Circular Polarization Based on Metasurface. IEEE T. Antenn. Propag..

[9] Paul R.W., James L.S., Alexander V.K., Vladimir M.S., Vladimir V.S., Friedrich S., Yuri A.Z., Robert K.D., Robert B. (2014). All-Dielectric Subwavelength Metasurface Focusing Lens. Opt. Express..

[10] Khorasaninejad M., Shi Z., Zhu A.Y., Chen W.T., Sanjeev V., Zaidi A., Capasso F. (2017). Achromatic Metalens over 60 nm Bandwidth in the Visible and Metalens with Reverse Chromatic Dispersion. Nano Lett..

[11] Huang L.L., Zhang S., Thomas Z. (2018). Metasurface Holography: From Fundamentals to Applications. Nanophotonics..

[12] Kim J., Yang Y.H., Badloe T., Kim I., Yoon G., Rho J. (2021). Geometric and Physical Configurations of Meta-atoms for Advanced Metasurface Holography. InfoMat.

[13] Sui S., Ma H., Wang J.F., Pang Y.Q., Feng M., Xu Z., Qu S.B. (2018). Absorptive Coding Metasurface for Further Radar Cross Section Reduction. J. Phys. D Appl. Phys..

[14] Dong G., Zhu S., He Y., Xia S., Zhang A., Wei X., Xu Z. (2018). Radar Cross Section Reduction Metasurface Based on Random Phase Gradients. Appl. Phys. B..

[15] Cui T.J. (2020). Electromagnetic Metamaterials: From Effective Media to Field Programmable Systems. Sci. Sin. Inform..

[16] Joy V., Dileep A., Abhilash P.V., Nair R.U., Singh H. (2021). Metasurfaces for Stealth Applications: A Comprehensive Review. J. Electron. Mater..

[17] Landy N.I., Sajuyigbe S., Mock J.J., Smith D.R., Padilla W.J. (2008). Perfect Metamaterial Absorber. Phys. Rev. Lett..

[18] Cai Y., Guo Y., Zhou Y.G., Huang X., Yang G., Zhu J. (2020). Tunable Dual-band Terahertz Absorber with All-dielectric Configuration Based on Graphene. Opt. Express..

[19] Yu K., Shen P., Zhang W., Xiong X., Zhang J., Liu Y. (2022). A Simple Structure for an Independently Tunable Infrared Absorber Based on a Non-Concentric Graphene Nanodisk. Materials.

[20] Muthukrishnan K., Narasimhan V. (2019). An Ultra-Thin Triple-Band Polarization-Independent Wide-Angle Microwave Metamaterial Absorber. Plasmonics.

[21] Sarkhel A., Bhadra S.R. (2017). Compact Quad-Band Polarization-Insensitive Ultrathin Metamaterial Absorber with Wide Angle Stability. IEEE Antenn. Wirel. Pr..

[22] Han X., Zhang Z., Qu X. (2021). A Novel Miniaturized Tri-band Metamaterial THz Absorber with Angular and Polarization Stability. Optik.

[23] Cheng R., Zhou Y., Wei R., Liu J., Liu H., Zhou X., Cai M., Pan X. (2022). Doubling and Tripling the Absorption Peaks of a Multi-band Graphene Terahertz Absorber. Diam. Relat. Mater..

[24] Hoque A., Tariqul Islam M., Almutairi A.F., Alam T., Jit Singh M., Amin N. (2018). A polarization independent quasi-TEM metamaterial absorber for X and Ku band sensing applications. Sensors.

[25] Moniruzzaman M., Islam M., Islam M., Chowdhury E., Rmili H., Samsuzzaman M. (2020). Cross coupled interlinked split ring resonator-based epsilon negative metamaterial with high effective medium ratio for multiband satellite and radar communications. Results Phys..

[26] Zu H., Wu B., Zhang Y., Zhao Y., Song R., He D. (2020). Circularly Polarized Wearable Antenna with Low Profile and Low Specific Absorption Rate Using Highly Conductive Graphene Film. IEEE Antenn. Wirel. Pr..

[27] Jiang S., Song R., Hu Z., Xin Y., Huang G., He D. (2022). Millimeter Wave Phased Array Antenna Based on Highly Conductive Graphene-assembled Film for 5G Applications. Carbon.

[28] Liu X., Song R., Fu H., Zhu W., Xiao Y., Zhang B., Wang S., He D. (2023). Anti-High-Power Microwave RFID Tag Based on Highly Thermal Conductive Graphene Films. Materials.

[29] Hui Y., Zu H., Song R., Fu H., Luo K., Tian C., Wu B., Huang G., Kou Z., Cheng X. (2023). Graphene-assembled Film-based Reconfigurable Filtering Antenna with Enhanced Corrosion-resistance. Crystals.

[30] Song R., Mao B., Wang Z., Hui Y., Zhang N., Fang R., Zhang J., Wu Y., Ge Q., Kostya S. (2023). Comparison of Copper and Graphene-Assembled Films in 5G Wireless Communication and THz Electromagnetic-Interference Shielding. PNAS.

[31] Fang S., Deng L., Zhang P., Qiu L., Xie H., Du J., Wang H., Zhao H. (2022). Dual-band Metamaterial Absorber with Stable Absorption Performance Based on Fractal Structure. J. Phys. D Appl. Phys..

[32] Song R., Zhao X., Wang Z., Fu H., Han K., Qian W., Wang S., Shen J., Mao B., He D. (2020). Sandwiched Graphene Clad Laminate: A Binder-Free Flexible Printed Circuit Broad for 5G Antenna Application. Adv. Eng. Mater..

[33] Ren Y., Ding J., Guo C., Qu Y., Song Y. (2017). Design of a Quad-Band Wide-Angle Microwave Metamaterial Absorber. J. Electron. Mater..

[34] Edries M., Mohamed H.A., Hekal S.S., El-morsy M.A., Mansour H.A. (2020). A New Compact Quad-Band Metamaterial Absorber Using Interlaced I/Square Resonators: Design, Fabrication, and Characterization. IEEE Access.

[35] Moniruzzaman M., Islam M.T., Hossain I., Soliman M.S., Samsuzzaman M., Almalki S.H. (2021). Symmetric Resonator Based Tunable Epsilon Negative Near Zero Index Metamaterial with High Effective Medium Ratio for Multiband Wireless Applications. Sci. Rep..

[36] Hakim M.L., Alam T., Islam M.T., Baharuddin M.H., Alzamil A., Islam M.S. (2022). Quad-Band Polarization-Insensitive Square Split-Ring Resonator (SSRR) with an Inner Jerusalem Cross Metamaterial Absorber for Ku- and K-Band Sensing Applications. Sensors.

[37] The L., Hong T., Dinh H., Xuan K., Son T., Hong L., Anh D., Dac T., Dinh L. (2020). Dual-Band Isotropic Metamaterial Absorber Based on Near-field Interaction in the Ku band. Curr. Appl. Phys..

[38] Hannan S., Islam M.T., Sahar N.M., Mat K., Chowdhury M., Rmili H. (2020). Modified-Segmented Split-Ring Based Polarization and Angle-Insensitive Multi-Band Metamaterial Absorber for X, Ku and K Band Applications. IEEE Access.

[39] Hannan S., Islam M.T., Almutairi A.F., Faruque M.R. (2020). Wide Bandwidth Angle- and Polarization-Insensitive Symmetric Metamaterial Absorber for X and Ku Band Applications. Sci. Rep..

[40] Nam M.H., Tung B.S., Khuyen B.X., Ha D.T., Ngoc N.V., Tran M.C., Le D.T., Lam V.D., Chen L., Zheng H. (2022). Graphene-Integrated Plasmonic Metamaterial for Manipulation of Multi-Band Absorption, Based on Near-Field Coupled Resonators. Crystals.

